# Bio-Protection as an Alternative to Sulphites: Impact on Chemical and Microbial Characteristics of Red Wines

**DOI:** 10.3389/fmicb.2020.01308

**Published:** 2020-06-16

**Authors:** Scott Simonin, Chloé Roullier-Gall, Jordi Ballester, Philippe Schmitt-Kopplin, Beatriz Quintanilla-Casas, Stefania Vichi, Dominique Peyron, Hervé Alexandre, Raphaëlle Tourdot-Maréchal

**Affiliations:** ^1^UMR Procédés Alimentaires et Microbiologiques, Université de Bourgogne Franche-Comté/AgroSup Dijon, Equipe VAlMiS (Vin, Aliment, Microbiologie, Stress), Institut Universitaire de la Vigne et du Vin Jules Guyot, Dijon, France; ^2^Centre des Sciences du Goût et de l’Alimentation, UMR 6265 CNRS, UMR 1324 INRA-Université de Bourgogne Franche Comté, Dijon, France; ^3^Analytical Food Chemistry, Technische Universität München, Munich, Germany; ^4^Research Unit Analytical BioGeoChemistry, Department of Environmental Sciences, Helmholtz Zentrum München, Neuherberg, Germany; ^5^Nutrition, Food Science and Gastronomy Department, INSA – XaRTA (Catalonian Reference Network on Food Technology), University of Barcelona, Santa Coloma de Gramenet, Spain

**Keywords:** wine bio-protection, metabolomic, phenolic and volatile compounds, sulphites, *Metschnikowia pulcherrima*

## Abstract

In wine, one method of limiting the addition of sulphites, a harmful and allergenic agent, is bio-protection. This practice consists of the early addition of microorganisms on grape must before fermentation. Non-*Saccharomyces* yeasts have been proposed as an interesting alternative to sulphite addition. However, scientific data proving the effectiveness of bio-protection remains sparse. This study provides the first analysis of the chemical and microbiological effects of a *Metschnikowia pulcherrima* strain inoculated at the beginning of the red winemaking process in three wineries as an alternative to sulphiting. Like sulphiting, bio-protection effectively limited the growth of spoilage microbiota and had no influence on the phenolic compounds protecting musts and wine from oxidation. The bio-protection had no effect on the volatile compounds and the sensory differences were dependent on the experimental sites. However, a non-targeted metabolomic analysis by FTICR-MS highlighted a bio-protection signature.

## Introduction

The large spectrum of action of sulfur dioxide (SO_2_), linked to its antioxidant, antimicrobial and antioxidasic activities, has justified its use in winemaking processes for many decades (Divol et al., 2012). However, in a context of societal concern regarding food and wine preservation, along with the quest for environmentally friendly and healthy production, reducing sulphite use now represents a major challenge for the wine industry (Salaha et al., 2008). Bio-protection is one of the alternatives recommended in the wine sector. This method consists in adding microorganisms on grape must before fermentation. Among these microorganisms, increasing attention has been focused on the selection of non-*Saccharomyces* (NS) yeast strains to develop new cultures capable of protecting grape musts and wines (Berbegal et al., 2018; Roudil et al., 2019).

Non-*Saccharomyces* yeasts have long been considered as spoilage microbiota (Fleet and Heard, 1993; Lambrechts and Pretorius, 2000; Ciani et al., 2010), but attention for this heterogeneous group of yeasts has renewed in recent years (Ciani and Picciotti, 1995; Ciani and Maccarelli, 1997; Egli et al., 1998; Henick-Kling et al., 1998; Rojas et al., 2001; Zohre and Erten, 2002; Fleet, 2003; Jolly et al., 2003a; Viana et al., 2008; Ciani et al., 2010; Comitini et al., 2011; Medina et al., 2013; Varela, 2016). NS yeasts are naturally dominant during the first steps of fermentation and give way to *Saccharomyces cerevisiae* yeasts for the core and final phases of alcoholic fermentation (Zott et al., 2008). Most studies have focused on the contribution of NS yeasts in sequential inoculation and their effect, along with those of *S. cerevisiae*, on the quality of the volatile fraction of wines (Garcia et al., 2002; Zohre and Erten, 2002; Jolly et al., 2003b; Anfang et al., 2009; Rodriguez et al., 2010; Sadoudi et al., 2012; Medina et al., 2013; Whitener et al., 2017). As a result, winemakers are now advised to use new NS starters to improve the organoleptic qualities of wine (Fleet, 2003; Ciani et al., 2010; Sadoudi et al., 2012; Beckner Whitener et al., 2015; Renault et al., 2015; Belda et al., 2017; Roudil et al., 2019).

In the context of bio-protection, the potential effect of the early addition of NS cultures on grapes and must appears more empiric. Many experiments have been carried out in wineries but very few scientific data proving the effectiveness of this practice are available. A single study on white winemaking showed that the early addition of a *Torulaspora delbrueckii* strain could provide a partial microbiological and chemical alternative to sulphites (Simonin et al., 2018). Indeed, it has been observed that the bio-protection strain added during the pre-treatment of the must (racking) induces the inhibition of spoilage microorganisms in the same way as sulphites. This “anti-microbial” effect is probably linked to interaction mechanisms between indigenous microorganisms and the bio-protection strain, including competition for nutrients and the production of killer toxins or other inhibitory compounds (Berbegal et al., 2018; Morata et al., 2019). The effect of the bio-protection strain against must oxidation appears more uncertain and appears directly related to the oxygen concentration measured in must. However, color differences observed in white must between sulphited and bio-protected modalities decreased over time and no significant difference was observed at the end of malolactic fermentation (Simonin et al., 2018).

Although bio-protection seems to be an effective strategy for partially or totally replacing sulphites when racking white musts, data is currently lacking on the impact of bio-protection during the pre-fermentation stages in red winemaking. The pre-fermentation maceration of grapes mainly permits the progressive extraction of various phenolic compounds such as anthocyanins and proanthocyanidins (Sacchi et al., 2005; Álvarez et al., 2006; Casassa and Harbertson, 2014; Setford et al., 2017). During this stage conducted at low temperature, grape musts have to be protected against chemical and microbial spoilages. This study conducted under real winemaking conditions compares the effect of adding a bioprotectant, a *M. pulcherrima* strain, as an alternative to sulphiting during pre-fermentation macerations. This species has been frequently found on grape berries (Pretorius, 2000; Prakitchaiwattana et al., 2004; Renouf et al., 2005). It has been reported that it is one of the NS yeasts with the most enzymatic activity (Rosi et al., 1994; Fernández-González et al., 2003; Rodriguez et al., 2004, 2010; González-Pombo et al., 2008; Zott et al., 2011; Theron et al., 2017) and is also able to inhibit the development of *Brettanomyces bruxellensis* yeasts under laboratory conditions (Oro et al., 2014). *B. bruxellensis* is one of the spoilage microorganisms, producing volatile phenols and negatively impacting wine properties (du Toit et al., 2005; Conterno et al., 2006; Oelofse et al., 2009; Agnolucci et al., 2010; Santos et al., 2011; Curtin et al., 2012; Longin et al., 2016). Other microorganisms such as acetic acid bacteria can also affect wine quality (Lonvaud-Funel, 1999; Du Toit, 2002; Bartowsky and Henschke, 2008; Mas et al., 2014).

In this context, this research is based on targeted microbiological and chemical analyses, including wine sensorial profiles. Moreover, for the first time, non-targeted analysis by Fourier transform ion cyclotron resonance mass spectrometry was applied to investigate the nature of bio-protected red wine chemistry.

## Materials and Methods

### Strains and Experiments

The bio-protection strain *M. pulcherrima* MCR 24 (AEB group – France), the *S. cerevisiae* strain Levulia PN^®^ (AEB group – France) and the *Oenococcus oeni* strain VP41 (Lallemand – France) were used. These strains were provided in dried form and rehydrated before inoculation according to the manufacturer’s instruction. These yeast strains are guaranteed to be very low sulphite producer (<10 mg/L).

Wine samples were produced with *Vitis vinifera* L. cv. Pinot Noir healthy grapes from Burgundy vineyards during the 2017 harvests. After harvesting, the grapes were processed in three different wineries named winery 1, winery 2, and winery 3. The initial sugar concentration in grape must was 219, 217, and 190 g/L, the Yeast Assimilable Nitrogen (YAN) was 180.2, 178.3, and 158.5 mg/L, the total acidity was 4.01, 4.17, and 4.20 mg/L (H_2_SO_4_) and the pH was 3.26, 3.32, and 3.30, respectively, for the tanks of winery 1, winery, and winery 3. No input was added to adjust the oenological parameters in grape musts. Two different modalities were conducted in a single replicate according to an identical protocol in each winery: the sulphite modality corresponding to the classical winemaking process and the bio-protection modality in which, sulphite was replaced by a bio-protection NS yeast.

For the bio-protection modality *M. pulcherrima* MCR 24 was used as bioprotectant. The *M. pulcherrima* strain were added to grapes during vatting, at the beginning of the pre-fermentation maceration. After rehydration, *M. pulcherrima* strain MCR 24 was added to 5 × 10^5^ CFU/mL corresponding to the bio-protected modality (BP modality). For the sulphite modality, 30 mg/L SO_2_ was added using a 5% (w/v) sulphite solution (S modality). Thus, macerations at 12°C were carried out in each winery in a 1,000 L a flat-bottomed stainless-steel tank. After 3 days of pre-fermentation maceration, *S. cerevisiae* strain Levulia PN^®^ was added for fermentations at 20°C in the three wineries (200 mg/L corresponding to 2 × 10^6^ CFU/mL). At the end of alcoholic fermentation, the wines were inoculated at 1 × 10^6^ CFU/mL with *O. oeni* VP41 to start malolactic fermentation. After the fermentations, all the wines were bottled with 30 mg/L of SO_2_ [5% (w/v)]. The sensorial analysis was carried out 2 months after bottling.

### Experimental Sampling

Samples were collected systematically for each winery: before the addition of the bio-protection strain or sulphites in the tank; during the cold pre-fermentation maceration (36 and 72 h); at mid-alcoholic fermentation (MAF); at the end of alcoholic fermentation (AF); at the end of malolactic fermentation (MLF). During the maceration and until the end of AF, the samples were taken after homogenization of the marc with the grape must at half-height of vat. After devatting and until the end of MLF, sampling was also carried out at half-height of vat. The enumeration of microorganisms was carried out until the end of AF. Analyses of anthocyanidin, proanthocyanidic, volatile, and non-volatile compounds, as well as sensory analyses were performed at the end of MLF.

### Detection of Microorganism Populations

The enumeration of NS yeast species and *S. cerevisiae* was carried out on Lysine agar medium and YPD agar medium, respectively, as described previously (Holm Hansen et al., 2001). *S. cerevisiae* was counted by subtracting the YPD agar medium colony count (enumeration of total yeasts) from the Lysine agar colony count. The incubation temperature was 28°C for 48 h for both agar media. *B. bruxellensis* populations were determined by plating on a specific medium composed of 10 g/L yeast extract, 20 g/L bacto-peptone, 20 g/L glucose, 0.1 g/L p-coumaric acid, 0.1 g/L ferulic acid, 0.03 g/L bromocresol green, 0.2 g/L chloramphenicol, 0.006% (w/v) cycloheximide (antifungal property), 20 g/L agar, and pH was adjusted to 4.8 as described by Gerbaux et al. (2009). The incubation temperature was 28°C for 7 days for this specific agar medium. Acetic bacteria were enumerated on a Mannitol agar medium composed of 25 g/L mannitol, 10 g/L yeast extract, 20 g/L agar, 10 mL/L Delvocid^®^ at 1% (w/v) (antifungal property) and 10 mL/L penicillin at 0.5% (w/v) (Gram-positive antibacterial property). The incubation temperature was 28°C for 72 h with 10% CO_2_. For the determination of the percentage of *M. pulcherrima* colonies present among NS yeasts, 30 colonies from each sample were isolated from Lysine agar medium. Each colony was grown in YPD liquid medium and conserved at −80°C in the presence of glycerol at 20% (v/v). Each colony was then identified by polymerase chain reaction-restriction fragment length polymorphism (PCR-RFLP) of internal transcribed spacers (ITS). The internal transcribed spacers ITS1/ITS4 and endonucleases CfoI/HaeIII were used to identify NS species yeasts, as described in Esteve-Zarzoso et al. (1999).

### Monitoring Fermentative Kinetics and Implanting Yeasts and Bacteria

In each winery, alcoholic fermentations (AF) and malolactic fermentations (MLF) were monitored by Fourier Transform Infrared Spectroscopy (FTIR Analysis by FOSS^®^). The concentrations of sugars and ethanol were determined during AF and the concentrations of ethanol (vol%), glucose/fructose (g/L), total acidity (g/L), pH, volatile acidity (g/L) and malic acid (g/L) were determined at the end of MLF. The implantation of the *S. cerevisiae* strain was controlled at mid-alcoholic fermentation by InterDelta analysis (Legras and Karst, 2003). The implantation of *O. oeni* strain was controlled at mid-malolactic fermentation by PCR Variable Number of Tandem Repeat (VNTR) with TR1 (Claisse and Lonvaud-Funel, 2014).

### Color Determination by Tristimulus Coordinates (L^∗^a^∗^b^∗^)

The color measurement of Tristimulus coordinates (L^∗^a^∗^b^∗^) was carried out at the end of MLF with a CM-5 Konica Minolta spectrophotometer. The visible absorption spectrum was recorded between 380 and 700 nm by reflectance to obtain Tristimulus values of L^∗^a^∗^b^∗^, using illuminant D65 and a standard observer (10° visual field) as references. The samples collected were centrifuged (3 min at 20°C, at 10,000 *g*) and 1 mL of each supernatant was transferred to a glass container (cell: 1mm). L^∗^a^∗^b^∗^ color space was used to quantify the color of the samples with L^∗^ for lightness, a^∗^ for redness and b^∗^ for yellowness. Three measurements were performed per sample.

### Analysis of Proanthocyanidins and Anthocyanins

The analyses were performed at the end of MLF.

#### Total Proanthocyanidin Concentration

Total proanthocyanidins are derived from Flavan-(3)-ol molecules [catechin, (2) – epicatechin]. In this method, these proanthocyanidins were converted into anthocyanins by heating in acidic and oxidizing media (Porter et al., 1985). For each wine, dilution to a fiftieth was performed in milliQ water. 2 mL of wine and 6 mL of reaction mixture [500 mL pure HCl (v/v), 500 mL butanol (n) (v/v), 150 mg of Fe_2_(SO_4_)_3_] were mixed with this diluted wine. Half of this mixture was placed in the dark (tube A). The other half of the mixture (tube B) was heated at 100°C for 30 min. The absorbance values were read at 550 nm. The variation of absorbance (Abs tube_B_ − Abs tube_A_) relative to a standard curve gave the total proanthocyanidin concentration.

[Totalproanthocyanidins](g/L)=(Abstube-BAbstube)A

×0.1736×50

#### Total Anthocyanin Concentration

Wine anthocyanins were determined on the basis of two properties due to their structures: color modification according to pH; the transformation into colorless derivatives under the action of certain reagents such as bisulphite ions. The variation of the absorbance value at 520 nm after the addition excess bisulphite ions was proportional to the anthocyanin content (Ribéreau-Gayon and Stonestreet, 1965) for each wine, 1 mL of wine, 1 mL of pure ethanol with 0.1% (v/v) HCl and 20 mL at 2% (v/v) HCl were mixed (mix 1). From this solution, 5 mL of mix 1 and 2 mL of Milli-Q water were added in the first tube (tube A). 5 mL of mix 1 and 2 mL of 15% (v/v) sodium bisulphite were added in the second tube (tube B), the tubes were placed in the dark for 30 min and the reading was taken at 520 nm. Absorbance variations (Abs tube_A_ − Abs tube_B_) relative to a standard curve gave the total anthocyanin concentration.

[Totalanthocyanins](g/L)=(Abstube-AAbstube)B×875*

875*:Slopeofthelinearcalibrationcurveobtainedwith

malvidin-3-glucoside.

#### Combined Anthocyanins Concentration

The red wine phenolic compounds were adsorbed by Polyvinylpolypyrrolidone (PVPP) (Glories, 1978). Afterward, only free anthocyanins were eluted with a specific solvent. 10 mL of PVPP soaked with distilled water was placed in a column. Then 5 mL of wine was introduced at the top of this column, which was then rinsed with 100 mL of distilled water. Free anthocyanins were eluted by the solvent (Ethanol/H_2_O/HCl − 70/30/0.1). The anthocyanin solution was then evaporated (maximum 40°C) and brought back to its initial volume of 5 mL with a synthetic wine [5 g/L tartaric acid, 0.89 g/L sodium hydroxide, 12% pure ethanol (v/v) and pH of 3.2]. The final eluate containing the free anthocyanins was finally dosed as explained in paragraph Total Anthocyanin Concentration to determine the concentration of combined anthocyanins.

[Combined⁢anthocyanins]⁢(g/L)=[Total⁢anthocyanins]-

[Free⁢anthocyanins]

#### Mean Degree of Polymerization (mDP)

The Mean Degree of Polymerization represents the degree of proanthocyanidin polymerization. To determine this value, the concentration of total proanthocyanidins is divided by the concentration of catechin. Dimethylaminocinnamaldehyde (DMACA) was used to determine the catechin concentration (Boukharta, 1988). DMACA induces the reaction between the nucleophilic part of the phloroglucinol nuclei and protonated aldehydes. This molecule reacts only with catechin monomers. To calculate the concentration in catechin, 0.5 mL of wine diluted to a fiftieth in Milli-Q water were mixed with 2 mL of reaction mixture 1 [300 mg of DMACA dissolved in 100 mL pure methanol (v/v) containing 3.5 mL at % (v/v) HCl and 77 mL pure methanol (v/v) with 23 mL at % (v/v) HCl]. Abs_wine_ was measured at 640 nm after 5 min. For the reagent blank, 0.5 mL of Milli-Q water was mixed with 2 mL of reaction mixture 1 and for the blank sample, 0.5 mL of wine diluted to a fiftieth in Milli-Q water was mixed with 2 mL of reaction mixture 2 [77 mL pure methanol (v/v) with 23 mL at % (v/v) HCl]. Abs_reagent blank_ and Abs_blank sample_ were measured at 640 nm.

mDP=[Total⁢proanthocyanidins]⁢(g/L)/[Catechins]⁢(g/L)

mDP=[Totalproanthocyanidins](g/L)/(0.0174×

(Abs-wineAbs-reagent⁢blankAbs)blank⁢sample×dilution)

### HS-SPME-GC/MS Analysis of Volatile Compounds

The analysis of volatile compounds was carried out at the end of MLF. Thirty-nine volatile compounds were assayed for each modality. 2 mL of wine was placed in a 10 mL vial, fitted with a silicone septum and placed in a silicon oil bath at 40°C, where the sample was maintained under magnetic stirring (300 rpm). After 10 min of sample conditioning, a DVB/CAR/PDMS fiber was exposed for 30 min to the sample headspace and immediately desorbed in the gas chromatograph injector.

Volatile compounds were analyzed by gas chromatography coupled to quadrupolar mass selective spectrometry using an Agilent 5973 Network detector (Agilent Technologies, Palo Alto, CA, United States). Analytes were separated on a Supelcowax-10 (Supelco) 60m × 0.25 mm I.D., 0.25 μm film thickness. The column temperature was held at 40°C for 10 min, increased to 200°C at 3°C/min, then to 150°C, then to 250°C at 15°C/min, and held for 5 min. The injector temperature was 260°C and the time of desorption of the fiber into the injection port was fixed at 5 min. The carrier gas was helium, at a flow rate of 1.5 mL/min. The temperature of the ion source was 175°C and that of the transfer line was 280°C. Electron impact mass spectra were recorded at 70 eV ionization energy, 2 scan/s. GC–MS analysis was performed in the complete scanning mode (SCAN), in the 30–300 u mass range.

The compounds were identified by comparing their mass spectra and retention times with those of standard compounds or with those available in the Wiley 6 mass spectrum library and in the literature, respectively. Response factors of volatile compounds were calculated using a calibration curve obtained by analyses of a hydroalcoholic solution (ethanol 10%, v/v) with different concentrations of reference compounds.

### FT-ICR-MS Metabolome Profiling

Direct-infusion FT-ICR mass spectra were acquired with a 12 Tesla Bruker Solarix FT-ICR mass spectrometer (Bruker Daltonics, Bremen, Germany). The samples were diluted 5:100 (v/v) with methanol (LC-MS grade, Fluka, Germany). The diluted samples were infused into the electrospray ion source at a flow rate of 2 μL/min. The settings for the ion source were: drying gas temperature 180°C, drying gas flow 4.0 L min^–1^, capillary voltage 3,600 V. The spectra were acquired with a time-domain of 4 megawords and 400 scans were accumulated within a mass range of m/z 92 to 1,000. A resolving power of 400,000 at m/z 300 was achieved.

The MS was first calibrated using arginine ion clusters (57 nmol/mL in methanol). Next, raw spectra were further internally calibrated using a reference list including known wine markers and ubiquitous fatty acids to achieve the best possible mass accuracy and precision among the samples. Raw spectra were post-processed by Compass DataAnalysis 4.2 (Bruker Daltonics, Bremen, Germany) and peaks with a signal-to-noise ratio (S/N) of at least 6 were exported to mass lists. All the exported features were aligned in a matrix containing averaged m/z values (maximum peak alignment window width: ±1 ppm) and corresponding peak intensities of all the samples analyzed. Only the m/z features of monoisotopic candidates and features with feasible mass defects were retained in the matrix. Due to the high resolving power of FT-ICR-MS, it is possible to calculate molecular formulas out of exact masses. A usual next step in data analysis is the annotation of compounds from databases.

### Sensorial Analysis

Twenty panelists recruited among the oenology students from the University of Burgundy took part in the sensorial analysis (11 women and 9 men; average age 24 years old). During their regular training in oenology they memorized aroma, taste and mouthfeel standards and also became familiar with the sensorial analysis of Burgundy red wines. They were not informed about the goal of the present study. The sensory analysis was divided into two sessions: free vocabulary generation and descriptive analyses using intensity scales.

During the first session the panelists generated a list of 18 attributes, and through consensus agreement developed a final list of descriptors. Arbitrarily, we removed the descriptors cited less than 3 times by the panel, i.e., 20% of the total list. The final list consisted of 1 visual, 9 aroma, and 3 taste and 5 mouthfeel attributes.

The second session consisted in a sensory descriptive analysis using the final list of descriptors obtained in the previous session. Panelists rated the wine, first for visual and aroma attributes, then for taste and mouthfeel attributes. Evaluation of all descriptors was performed on a ten-points intensity scale, from 0 “absent” to 9 “very strong.”

For both sessions, the samples (30 mL) were served in a transparent ISO glass covered with a plastic cup cover (PL2 model; Solo Cup Co.). Samples were presented in three series (one by winery) of two samples (S and BP). Presentation orders were balanced within series but the order of the series was the same for every panelist.

### Statistical Analysis

Three replicates of analyses for each modality were used for microorganism population enumerations, proanthocyanidins – anthocyanins and classical oenological parameters. The pair-wise comparison approach (Tukey test) was used to compare data from both modalities.

For FT-ICR-MS, all further data processing was done in R Statistical Language (version 3.4.1). Principal Component Analysis (PCA), Hierarchical Cluster Analysis (HCA), and analysis of variance (ANOVA) were performed using Perseus 1.5.1.6 (Max Planck Institute of Biochemistry, Germany). For HCA, Euclidean distance and average linkage were chosen and for ANOVA, the threshold *p*-value was 0.05.

Samples at the end of MLF were analyzed six times for volatile compounds. Thirty-nine compounds were assayed and subjected to a pair-wise comparison approach. The significant data were then exploited as PCA.

Concerning the sensory analysis, a two-factor (panelist and treatment) analysis of variance (ANOVA) was carried out for each winery on the scores collected in session 2. *Post hoc* mean comparison Tukey test (α = 5%) was performed on the descriptors with significant treatment effect.

An Excel spreadsheet was used to create the graphs.

## Results and Discussion

### Dynamics of Microorganism Populations on Must and Alcoholic Fermentation

The potential effect of a bio-protection strain, used as an alternative to the antimicrobial action of sulphites, was first investigated. Before maceration, the populations of NS, acetic bacteria and *B. bruxellensis* were heterogeneous between the different wineries, as described in the literature (Barata et al., 2012). In the three wineries, the NS population levels were in the order of 10^4^ CFU/mL with a percentage of native *M. pulcherrima* species of 33% in winery 1, 7% in winery 2 and 10% in winery 3 (Table 1). The presence of potential spoilage agents such as acetic bacteria and *B. bruxellensis* yeasts was also noted. The concentrations of these microorganisms were highly variable between wineries, with relatively high levels of acetic bacteria in the three wineries and a fairly high concentration of *B. bruxellensis* with 2.03 × 10^3^ CFU/mL in winery 3. This microbiota can negatively affect wine by producing undesirable acetic acid and volatile phenols (Renouf et al., 2007).

**TABLE 1:** Numeration of the different populations during the winemaking process: NS yeasts, *S. cerevisiae* yeasts, *B. bruxellensis* yeasts, acetic bacteria on different agar media for BP and S modalities at different winemaking times in wineries 1, 2, and 3.

	**Winery 1**							
**Step of winemaking process**	**NS yeasts (CFU/mL) - (% *M. pulcherrima Mp*)**		***S. cerevisiae* yeasts (CFU/mL)**		***B. bruxellensis* yeasts (CFU/mL)**		**Acetic acid bacteria (CFU/mL)**	
				
	**BP modality**	**S modality**	**BP modality**	**S modality**	**BP modality**	**S modality**	**BP modality**	**S modality**
Maceration before bio-protection or sulphite addition	4.33 × 10^4^ – **(33% *Mp*)**		ND		<3.00 × 10^1^		2,33 × 10^2^	
During maceration (36 h)	6.00 × 10^5 a^ – **(100% *Mp*)**	3.67 × 10^3^ ^*b*^ – **(0% *Mp*)**	ND	ND	<3.00 × 10^1^ ^*a*^	<3.00 × 10^1^ ^*a*^	6.67 × 10^1^ ^*a*^	ND ^*a*^
End of maceration before *S. cerevisiae* addition (72 h)	7.00 × 10^5 a^ – **(100% *Mp*)**	1.00 × 10^2^ ^*b*^ – **(57% *Mp*)**	ND	ND	<3.00 × 10^1 a^	3.33 × 10^1^ ^*a*^	7.00 × 10^1^ ^*a*^	ND ^*a*^
End of AF	ND	ND	8.33 × 10^6 a^	6.67 × 10^6 a^	ND	ND	ND	ND

	**Winery 2**							
	
**Step of winemaking process**	**NS yeasts (CFU/mL) - (% *M. pulcherrima Mp*)**		***S. cerevisiae* yeasts (CFU/mL)**		***B. bruxellensis* yeasts (CFU/mL)**		**Acetic acid bacteria (CFU/mL)**	
				
	**BP modality**	**S modality**	**BP modality**	**S modality**	**BP modality**	**S modality**	**BP modality**	**S modality**

Maceration before bio-protection or Sulphite addition	9.67 × 10^4^ – **(7% *Mp*)**		ND		6.67 × 10^0^		5,00 × 10^3^	
During maceration (36 h)	4.00 × 10^5 a^ – **(63% *Mp*)**	2.33 × 10^5 a^ – **(0% *Mp*)**	ND	ND	1.47 × 10^2^ ^*a*^	2.87 × 10^2^ ^*a*^	3.67 × 10^4^ ^*a*^	4.33 × 10^3^ ^*a*^
End of maceration before *S. cerevisiae* addition (72 h)	4.00 × 10^5 a^ – **(10% *Mp*)**	2.00 × 10^4^ ^*b*^ – **(0% *Mp*)**	ND	ND	8.00 × 10^1 b^	7.10 × 10^2^ ^*a*^	7.00 × 10^5^ ^*a*^	3.33 × 10^4^ ^*b*^
End of AF	2.63 × 10^3 a^ – **(0% *Mp*)**	1.03 × 10^3 b^ – **(0% *Mp*)**	7.64 × 10^5 a^	1.07 × 10^6 a^	8.00 × 10^1 a^	<3.00 × 10^1 a^	6.33 × 10^3^ ^*b*^	1.83 × 10^4^ ^*a*^

	**Winery 3**							
	
**Step of winemaking process**	**NS yeasts (CFU/mL) - (% *M. pulcherrima Mp*)**		***S. cerevisiae* yeasts (CFU/mL)**		***B. bruxellensis* yeasts (CFU/mL)**		**Acetic acid bacteria (CFU/mL)**	
				
	**BP modality**	**S modality**	**BP modality**	**S modality**	**BP modality**	**S modality**	**BP modality**	**S modality**

Maceration before bio-protection or Sulphite addition	9.00 × 10^4^ – **(10% *Mp*)**		ND		2.03 × 10^3^		4,00 × 10^2^	
During maceration (36 h)	1.90 × 10^6 a^ – **(57% *Mp*)**	5.00 × 10^3^ ^*b*^ – **(3% *Mp*)**	ND	ND	4.37 × 10^3^ ^*a*^	5.00 × 10^2^ ^*b*^	1.30 × 10^4^ ^*a*^	1.23 × 10^4^ ^*a*^
End of maceration before *S. cerevisiae* addition (72 h)	1.13 × 10^7 a^ – **(21% *Mp*)**	2.87 × 10^3^ ^*b*^ – **(18% *Mp*)**	ND	ND	4.23 × 10^3 a^	3.33 × 10^2^ ^*a*^	1.70 × 10^4^ ^*a*^	5.00 × 10^3^ ^*b*^
End of AF	1.07 × 10^3 b^ – **(7% *Mp*)**	4.63 × 10^3 a^ – **(40% *Mp*)**	7.99 × 10^5 b^	3.00 × 10^6 a^	5.67 × 10^2^ ^*a*^	4.67 × 10^2^ ^*a*^	ND ^*b*^	7.62 × 10^2^ ^*a*^

*% Mp (bold values) represents the percentage of M. pulcherrima species. ^*a*,*b*^Significant differences by the Tukey test were found between the two modalities of the same winery at a given winemaking time. ND, not detected.*

The effect of sulphite addition was observed in wineries 1 and 3, with a large reduction (about 10 times lower) of NS populations during the maceration (Table 1). In winery 2, the sulphites were less effective in reducing natural microbiota except for native *M. pulcherrima* yeasts. Concerning the effect of SO_2_ on spoilage microbiota, this evolution was totally dependent on the experimental sites. Sulphite addition eliminated acetic bacteria in winery 1 while these microorganisms were still present in large quantities in winery 2 and winery 3, with 4.33 × 10^3^ and 1.23 × 10^4^ CFU/mL, respectively. For *B. bruxellensis* populations, the same observation was made, with only a slight decrease in winery 3. The effect of SO_2_ on the NS yeasts has been described previously in the literature (Henick-Kling et al., 1998). The antimicrobial action of sulphites on different microorganisms was clearly dependent on the starting must and the combination of free sulphite with must compounds (Divol et al., 2012).

After the inoculation of *M. pulcherrima* MCR24 (5.00 × 10^5^ CFU/mL), there was an increase in the population of NS yeasts of about one log of CFU/mL with 100% of *M. pulcherrima* in winery 1, 63% in winery 2 and 57% in winery 3 (Table 1). Higher levels of NS yeasts combined with the percentages of *M. pulcherrima* suggested the good implantation of the added bio-protection strain. Indeed, no molecular identification techniques have been developed. Although the population of the *M. pulcherrima* species decreases during pre-fermentation maceration, the use of this NS strain is accompanied by a limited development of spoilage microbiota. A slight increase of *B. bruxellensis* species was observed in all the wineries and the evolution of acetic bacteria depended on the experimental sites, with an increase in wineries 2 and 3 and a decrease in winery 1 compared to the starting musts. If we compare these results with those of S modalities, few significant differences were found between modalities. However, in winery 3 we can note a cell multiplication of *M. pulcherrima* with ten times more cells between the middle and the end of maceration compared to the other wineries. Furthermore, *M. pulcherrima* cells were still detected at the end of the alcoholic fermentation. (Table 1), which may explain the volatile acidity concentrations observed at the end of AF (0.42 g/L) compared to the other wineries (winery 1: 0.29 g/L and winery 2: 0.38 g/L) (Table 2). This observation correlates with the study of Sadoudi et al. (2017) showing that the cohabitation of these two species with populations above 10^6^ CFU/mL could lead to higher volatile acidity production by *S. cerevisiae*. After maceration, musts were inoculated with the same *S. cerevisiae* strain. The strain was successfully implanted for all fermentations (Supplementary Figure S1) and fermentative kinetics were similar between BP and S modalities for each winery (data not shown). At the end of AF, the development of spoilage microorganisms was limited whatever the modality. The action of the bio-protection strain on other microorganisms could be due to a biomass effect and selective pressure on grape must (Nissen et al., 2003). *M. pulcherrima* was added to 5.00 × 10^5^ CFU/mL and represented between 5- and 12-fold the initial population in must. Such a high level of *M. pulcherrima* could lead to niche pre-emption *via* a rapid resource depletion, as reported before by Dhami et al. (2016). Indeed, competition between species for oxygen, lipids, nitrogen in must and wine has been reported before (Morales et al., 2015; Gobert et al., 2017). The production of pulcherriminic acid may also explain the inhibition of spoilage microorganisms (Oro et al., 2014). Indeed, the production of pulcherriminic acid has been described as an iron chelator (pulcherrimin) which inhibits the development of some strains of *B. bruxellensis* that need iron for their own development (Oro et al., 2014). The common point of all the wineries is that the addition of bio-protection increased the concentration of NS yeast and the percentage of *M. pulcherrima* yeast suggesting its good implantation. This high percentage of *M. pulcherrima*, closely linked to the bio-protection strain inoculation, could explain the limited growth of spoilage microorganisms such as B. bruxellensis yeasts and acetic acid bacteria until the end of AF. Only a control modality (without bio-protection and sulphites) would have confirmed this impact. Nevertheless, these data strongly supported the hypothesis of what had already been previously observed in white winemaking (Simonin et al., 2018), with the control of spoilage microorganisms when adding bio-protection at the beginning of the winemaking process.

**TABLE 2:** Oenological parameters were measured at the end of MLF by FTIR for all modalities.

**Modalities**	**Ethanol (vol%)**	**[Glucose/fructose] (g/L)**	**[Total acidity] (g/L H_2_SO_4_)**	**pH**	**[Volatile acidity] (g/LH_2_SO_4_)**	**[Malic acid] (g/L)**
Winery 1 –BP modality	13.05^*a*^	<1.0^*a*^	3.50^*a*^	3.52^*a*^	0.29^*a*^	0.0^*a*^
Winery 1 –S modality	13.20^*a*^	<1.0^*a*^	3.55^*a*^	3.47^*a*^	0.25^*a*^	0.0^*a*^
Winery 2 –BP modality	13,00^*a*^	<1.0^*a*^	3.40^*a*^	3.79^*a*^	0.38^*a*^	0.1^*a*^
Winery 2 –S modality	13,00^*a*^	<1.0^*a*^	3.70^*a*^	3.63^*a*^	0.36^*a*^	0.1^*a*^
Winery 3 –BP modality	11.40^*a*^	<1.0^*a*^	3.40^*a*^	3.50^*a*^	0.42^*a*^	0.1^*a*^
Winery 3 –S modality	11.22^*a*^	<1.0^*a*^	3.20^*a*^	3.60^*a*^	0.36^*a*^	0.1^*a*^

*^*a*,*b*^The values for ethanol (vol%), glucose/fructose (g/L), total acidity (g/L), pH, volatile acidity (g/L) malic acid (g/L) were statistically compared within the same winery by the Tukey test.*

### Analysis of Oenological Parameters, Proanthocyanidins and Anthocyanins

**FIGURE 1:**
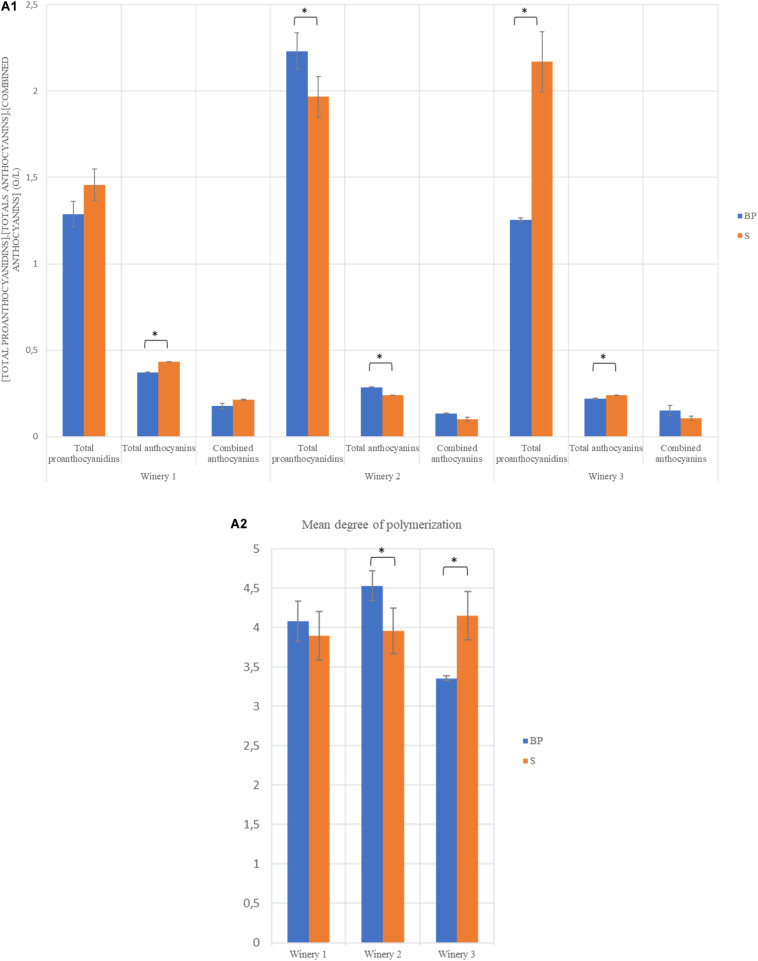
Histogram representing the concentration of total proanthocyanidins, total anthocyanins, combined anthocyanins in g/L **(A1)** and the mean degree of polymerisation (mDP) **(A2)** at the end of MLF in each winery. ^∗^The Tukey test found significant differences between the two modalities of the same winery.

After AF, wines were inoculated with the same *O. oeni* strain. The implantation of this strain was successful in the three wineries (Supplementary Figure S2). The analyses of wines after MLF demonstrated that the addition of the bio-protection strain or sulphites during pre-fermentation stage did not impact the oenological parameters at the end of MLF (Table 2). The values for these parameters were statistically compared within the same winery and no significant differences were noted between modalities. The data presented in Figure 1 shows that the total proanthocyanidin concentration was significantly higher for the BP modality in winery 2, with 2.23 g/L compared to 1.97 g/L for the S modality, whereas the contrary was observed in winery 3, with 1.25 g/l for the BP modality compared to 2.17 g/L for the S modality. Significant differences were observed for total anthocyanin concentrations, with a higher content for S modalities in wineries 1 and 3, with 0.43 and 0.24 g/L, respectively, compared to 0.37 and 0.22 g/L, respectively, for BP modalities (Figure 1-A1). The contrary was observed in winery 2 with 0.29 g/L for the BP modality and 0.24 g/L for the S modality. No significant difference was detected for combined anthocyanins. Regarding the mean Degree of Polymerization (mDP), this value was significantly higher for the BP modality in winery 2, while it was the opposite in winery 3 (Figure 1-A2). Sulphites play an antioxidant and an extractant role with respect to proanthocyanidins and anthocyanins. Moreover, they are known to prevent or to slow down polymerization reactions (Divol et al., 2012). We hypothesized that the replacement of sulphites by a bio-protection strain affects proanthocyanidin and anthocyanin levels and leads to a higher degree of polymerization. But no trend emerged, suggesting that the significant differences observed were mainly dependent on the initial phenolic composition of the matrix.

### FT-ICR-MS Analysis

The performance of non-targeted metabolomics, considered here as comprehensive analyses of chemical diversity, is unprecedented (Roullier-Gall et al., 2014, 2017). The wine metabolome consists of many thousands of single compounds and ultra-high-resolution mass spectrometric methods are essential to characterize them. Fourier Transform Ion Cyclotron Resonance Mass spectrometry (FT-ICR-MS) is the most efficient mass spectrometric method for achieving ultra-high mass resolution, sensitivity and accuracy, and is capable of efficiently detecting thousands of metabolites.

**FIGURE 2:**
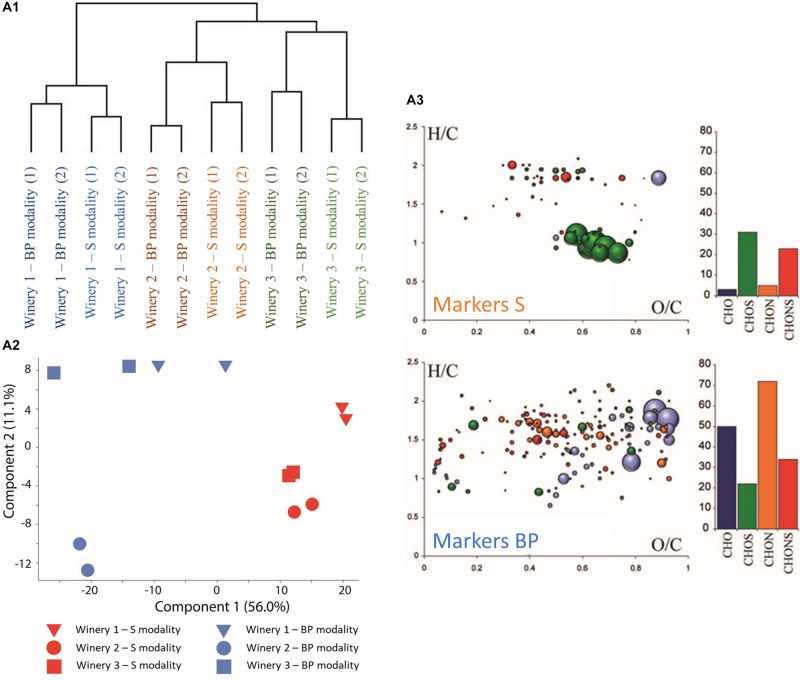
**(A1)** HCA representing all the compounds found by FTICR-MS analysis for all the wineries. Samples were analyzed twice at the end of MLF. **(A2)** Principal component analysis (PCA) representing significantly different compounds in Mp and S control modalities. **(A3)** Van Krevelen diagram for compounds specific to BP modalities (Markers BP) and S modalities (Markers S) with H/C on O/C.

Unequivocal elemental formulas can be achieved for each mass detected but analyzing the structural information of the elemental formulas identified remains a challenge due to the possible presence of several isomers. Therefore, the use of metabolite databases such as KEGG, HMDB, YMDB, or Lipidmaps is helpful for pointing to hypotheses and suggestions for possible and relevant chemical structures behind the metabolic formula identified. Here, FT-ICR-MS analysis was performed at the end of MLF. We recorded the molecular composition in a non-targeted approach using direct infusion and a complex pool of thousands of distinct ion signals was observed. About 12,000 peaks were found in the samples. The instrument’s high resolving power and mass accuracy enabled assigning the individual mass peaks to their corresponding unique molecular compositions. Thus, 7,635 of the 12,000 peaks were assigned to molecular formulas based on carbon, hydrogen, oxygen, nitrogen, and sulfur atoms. Hierarchical cluster analysis (HCA) (Figure 2-A1) based on the 7,635 annotated compounds highlighted a clear distinction between the wineries independently of bio-protection or sulphite use, confirming the geographical origin and different matrix impacts (Roullier-Gall et al., 2014). According to the cluster analysis, winery 1 seemed to be the most different compared to wineries 2 and 3 (Figure 2-A1). A closer look at the HCA within each winery, allows distinguishing the BP and S modalities. According to HCA, the BP and S modalities are closer in winery 1 than in winery 2 and 3. Venn diagrams on the annotated compounds (Supplementary Figure S3), confirmed the closest similarity between wineries 2 and 3 with 849 common compounds, although most of the compounds detected were present in all three wineries (5,144 common compounds present in all the wineries). Regarding chemical species, the wine obtained from the BP and S modalities appeared to be closer in composition when compared to the impact of the wineries. Since the origin of the wineries had the highest impact on wine composition, the differences between the modalities within each winery was first highlighted using Venn and van Krevelen diagrams (Supplementary Figures S3, S4). The first interesting fact is that the majority of annotated compounds were common to the modalities employed in the three wineries. Indeed, more than 75% of annotated compounds were detected in both the BP and S modalities. The second point of note is that the unique compounds detected in the BP and S modalities differed according to the winery. Thus, for winery 1, the unique compounds in the S modality were mostly CHO compounds (in blue) while the unique compounds in the BP modality for wineries 2 and 3 were mostly CHNOS compounds (in red). The same finding was obtained for the BP modality, where the unique compounds are mostly CHON compounds, follow by CHONS compounds for wineries 1 and 3. The ratio was reversed for winery 2 with a higher number of CHONS followed by CHON compounds. In order to assess the impact of using either bio-protection or sulphites on the chemical compositions of the wines, FT-ICR-MS data were statistically processed to extract common markers independently of the winery (ANOVA with *p*-values < 0.05) to each group (BP versus S modalities). The significant molecular formulas were then represented by principal component analysis (PCA) and van Krevelen diagrams (Figures 2-A2,A3). Van Krevelen diagrams provide a visual representation of elemental composition distribution (CHO, CHOS, CHON, and CHNOS) according to hydrogen to carbon and oxygen to carbon atomic ratios. Three hundred and eighty specific compounds were extracted for BP modalities and one hundred and fifty-eight for S modalities (Figure 2-A3). Based on the extracted specific compounds, the PCA clearly discriminated BP and S modalities. The first component C1, explaining 56.0% of the variability, mainly differentiated BP modalities from S modalities while the second, C2 explaining 11.1% of the variability, highlighted the difference between the wineries (Figure 2-A2).

Van Krevelen diagrams showing specific markers for BP and S modalities complete this analysis (Figure 2-A3). CHO and CHON containing compounds were in higher proportion in the specific masses for BP modalities (Figure 2-A3 Markers BP) compared to S modalities, whereas sulfur containing compounds (CHOS and CHONS) were represented more in the specific masses for S modalities (Figure 2-A3 Markers S). The higher number of sulfur containing compounds in S modalities could be explained by the addition of 30 mg/L of sulphite at vatting. Added sulphites mainly combine with other compounds in the must, as shown previously (Roullier-Gall et al., 2017), which could explain the higher number of sulfur containing compounds. On the other hand, the higher proportion of CHO and CHON in the BP modalities could be explained in part by the production of certain molecules (amino acids, proteins, etc.) by the *M. pulcherrima* strain (Theron et al., 2017). Although this number of molecules remained very low, it could be considered as a signature of bio-protection addition.

### Wine Volatile Compounds

**FIGURE 3:**
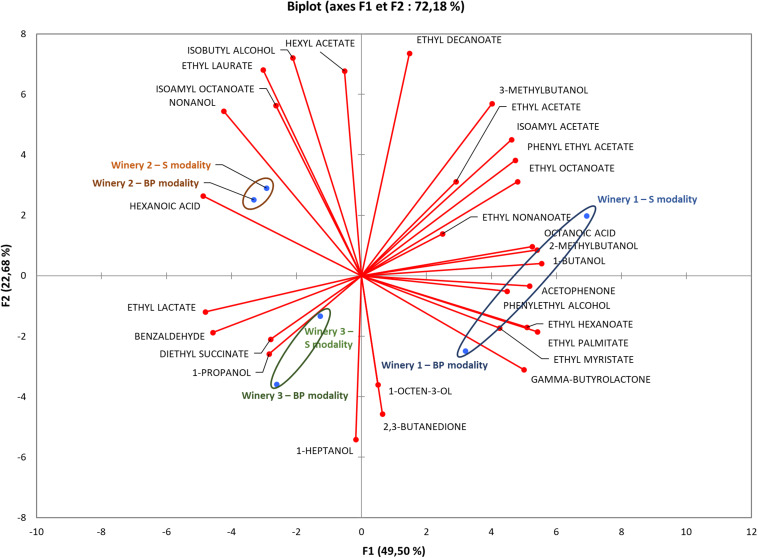
Principal component analysis profile discriminating wineries and volatile compounds. Analysis of volatile compounds was carried out at the end of MLF for all wineries. Significant differences in volatile compound concentrations were used to perform this analysis.

Thirty-nine volatile esters and higher alcohol compounds were quantified in the wines (Supplementary Table S2). PCA showed the main differences acquired in our dataset (Figure 3) and highlighted that the differences in volatile compounds were greater between wineries compared to the modalities, reflecting matrix effects and supporting the findings of the FT-ICR-MS analysis. The presence of *M. pulcherrima* during pre-fermentation grape maceration did not impact the volatile compound compositions of wines. The populations of *S. cerevisiae* were highest during alcoholic fermentation in all the wineries, with approximatively 10^8^ CFU/mL (data not shown). Thus, the impact of bio-protection remained weak, whereas the *S. cerevisiae* strain, which was well established in all the wineries, contributed strongly to various volatile compounds in the different wines.

### Sensorial Analysis

**FIGURE 4:**
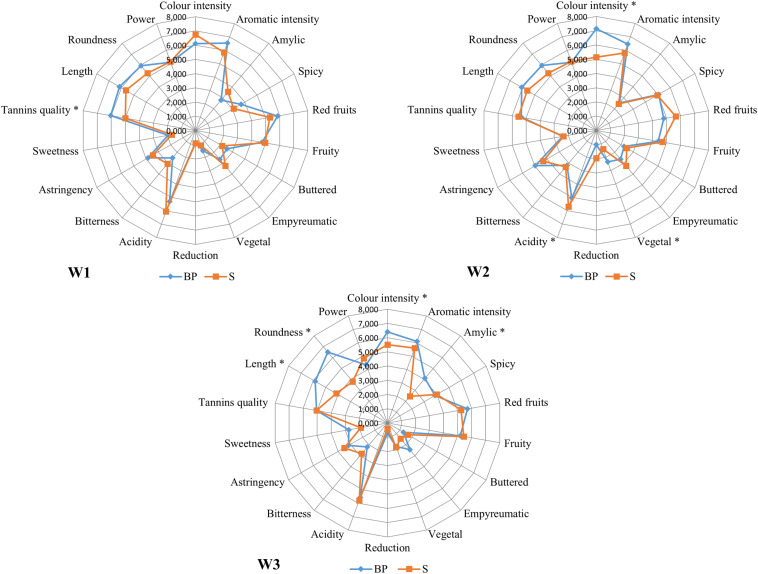
Sensory profiles of wine from winery 1 **(W1)**, winery 2 **(W2)**, and winery 3 **(W3)**. Orange lines correspond to S modalities and blue lines correspond to BP modalities. ^∗^Significant differences (Tukey test, α = 5%).

Free vocabulary generation followed by sensory descriptive analysis highlighted the different descriptors of the wines produced by bio-protection and sulphiting. The Figure 4 shows the average scores for each attribute and each sample for W1, W2, and W3 separately. Very few attributes showed significant effects between treatments and none of the effects were common to the three wineries. In fact, nearly all of them were specific to one winery excepted color intensity. Indeed, the quality of the tannins was highest in the BP modality for winery 1 (Figure 4-W1). For winery 2 (W2), the profiles also highlighted a difference in acidity in favor of the S modality. The vegetal descriptor was described as more intense in the BP modality and the color intensity was more pronounced in the BP modality (Figure 4-W2). For winery 3 (Figure 4-W3), roundness, length and amylic were significantly more intense in the BP modality. The color intensity was also more pronounced in the BP modality (Figure 4-W3).

It is interesting to note that the only specific attribute was color intensity which was highest in the BP modality for wineries 2 and 3. These differences were confirmed by the Tristimulus coordinates (L^∗^a^∗^b^∗^) (Supplementary Table S1). Globally speaking, the results of the sensory analysis did not pinpoint any sensory signature of the wines produced from BP modalities, which suggest that the differences found resulted from a matrix effect. Moreover, no descriptor related to *B. bruxellensis* yeasts, acetic or lactic acid bacteria, or related to wine oxidation were found for the complete list of free vocabulary generation before removing 20%. No negative organoleptic deviance was observed with the addition of the bio-protection strain.

## Conclusion

We reported for the first time the effect of bio-protection on red grape varieties during pre-fermentation maceration, used as an alternative to sulphites. From the microbiological standpoint, the lack of a control modality, without the addition of bio-protection and sulphites, did not confirm the true effect of *M. pulcherrima* strain biomass on spoilage microorganisms (*B. bruxellensis* yeasts and acetic acid bacteria). However, the results obtained are promising as they observed that under the condition tested, the use of *M. pulcherrima* is accompanied by a limited growth of spoilage microbiota. Additional studies will still be required to confirm the impact of the addition of bio-protection on spoilage microorganisms after AF, allowing to secure the stability of the wine before bottling. Contrary to what might have been expected, there was no effect related to bio-protection on the phenolic composition of wines, since the matrix effect was greater than the bio-protection effect. In contrast, wines produced from bio-protected or sulphited musts had different metabolic signatures, probably reflecting the production of specific metabolites by *M. pulcherrima* or the presence of chemical adducts due to sulphites. The metabolomics approach carried out by FT-ICR-MS analyses revealed statistical discriminations, contrary to analyses of volatile compounds and conventional oenological analyses. In the future, this approach will allow us to focus on these specific metabolites in order to further investigate *M. pulcherrima* metabolism during pre-fermentation step.

No impact of bio-protection on volatile compound composition was observed and sensory differences were specific to each winery and not related to volatile compounds. This was probably due to the fact that the low temperature value of grape must during pre-fermentative maceration (12°C) limits the growth of the *M. pulcherrima* yeast added. The bioprotectant population was a 100-fold lower than the *S. cerevisiae* population present in both modalities. The impact of the bio-protection strain was therefore limited on the production of volatile compounds during alcoholic fermentation and, therefore, on the sensory analyses.

This work demonstrated that bio-protection is a credible alternative to sulphites for the Pinot Noir grape varieties under the experimental conditions of this work. This strategy was all the more interesting because it did not drastically modify the sensory profile of wine.

## Data Availability Statement

All datasets generated for this study are included in the article/Supplementary Material.

## Author Contributions

SS, RT-M, and HA conceived and designed the study, performed experiments in real winemaking conditions, and identified microorganisms and monitoring fermentative kinetics. SS and DP analysis of color, proanthocyanidins, and anthocyanins. CR-G, PS-K, SS, HA, and RT-M metabolome profiling. SV, BQ-C, SS, HA, and RT-M determination of the concentration of volatile compounds in the wine and analysis. SS, JB, HA, and RT-M study of sensorial analysis. SS drafted the manuscript. SS, RT-M, HA, CR-G, JB, DP, PS-K, SV, and BQ-C corrected and refined the manuscript. All authors read and approved the final manuscript.

## Conflict of Interest

The authors declare that the research was conducted in the absence of any commercial or financial relationships that could be construed as a potential conflict of interest.
