# Supplementary material for: Bio-Protection as an Alternative to Sulphites: Impact on Chemical and Microbial Characteristics of Red Wines
Source: Front Microbiol. 2020 Jun 16;11:1308. doi: 10.3389/fmicb.2020.01308 (PMC7308991; doi:10.3389/fmicb.2020.01308)
Supplement: Supplementary file 1 [file Data_Sheet_1.PDF]

Supplementary Material

Bio-protection as an alternative to sulphites: impact on chemical and microbial characteristics of red wines

Scott Simonin[1*], Chloé Roullier-Gall[1], Jordi Ballester[2], Philippe Schmitt-Kopplin[3,4], Beatriz Quintanilla-Casas[5], Stefania Vichi[5], Dominique Peyron[2], Hervé Alexandre[1], Raphaëlle Tourdot-Maréchal[1]

*** Correspondence:** scott.simonin@laposte.net

1 Supplementary Figures and Tables

1.1 Supplementary Figures

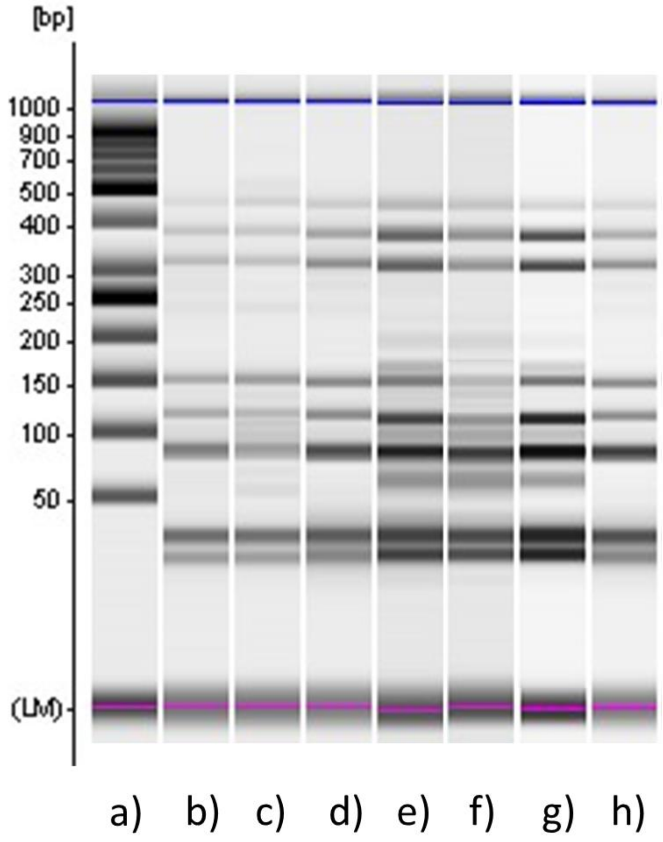

Supplementary Figure 1. (Fig S1) Control of the implantation of Active Dry Yeast (ADY) at mid-alcoholic fermentation. The method used is the Interdelta PCR for *Saccharomyces cerevisiae* strain characterization. The result of these amplifications is analyzed using MCE-202 MultiNA. a) corresponding to Ladder 50 ppb, b) to ADY, c) to BP modality in Winery 1, d) to S modality in Winery 1, e) to BP modality in Winery 2, f) to S modality in Winery 2 and g) to BP modality in Winery 3, h) to S modality in Winery 3.

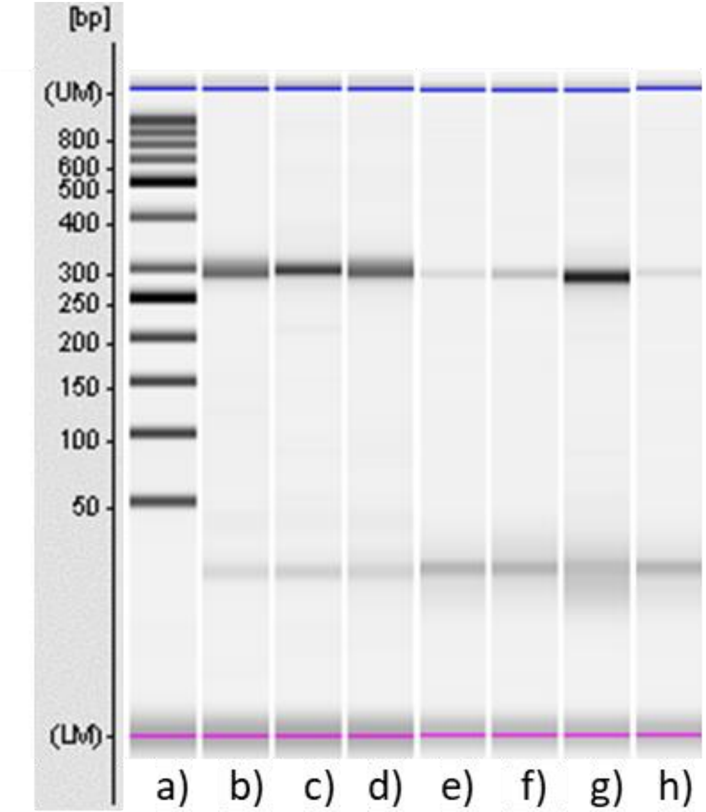

Supplementary Figure 2. (Fig S2) Control of the implantation of *O. oeni* strain at mid- malolactic fermentation. The method used is the PCR VNTR with TR1 with for *Oenococcus oeni* strain characterization. The result of these amplifications is analyzed using MCE-202 MultiNA. a) corresponding to Ladder 50 ppb, b) to *O. oeni* reference, c) to BP modality in Winery 1, d) to S modality in Winery 1, e) to BP modality in Winery 2, f) to S modality in Winery 2 and g) to BP modality in Winery 3, h) to S modality in Winery 3.

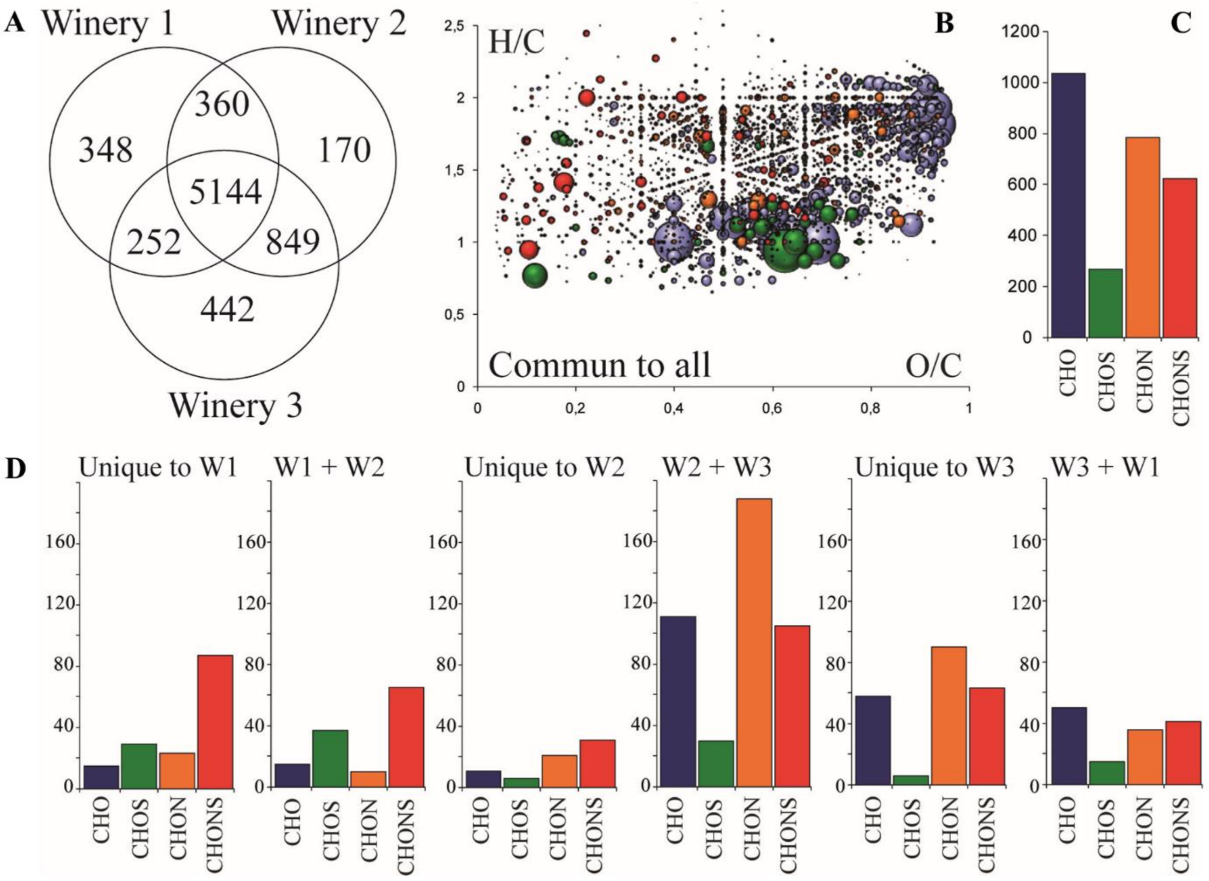

Supplementary Figure 3. (Fig S3) Venn diagrams representing all annotated common and uncommon compounds in three wineries (A). Van Krevelen diagram (B) (H/C vs. O/C atomic ratio) and histogram (C) (CHO, CHOS, CHON and CHONS) representing all annotated compounds. Van Krevelen histogram representing all compounds Unique to W1, W1+W2, Unique to W2, W2+W3, Unique to W3 and W3+W1 (D).

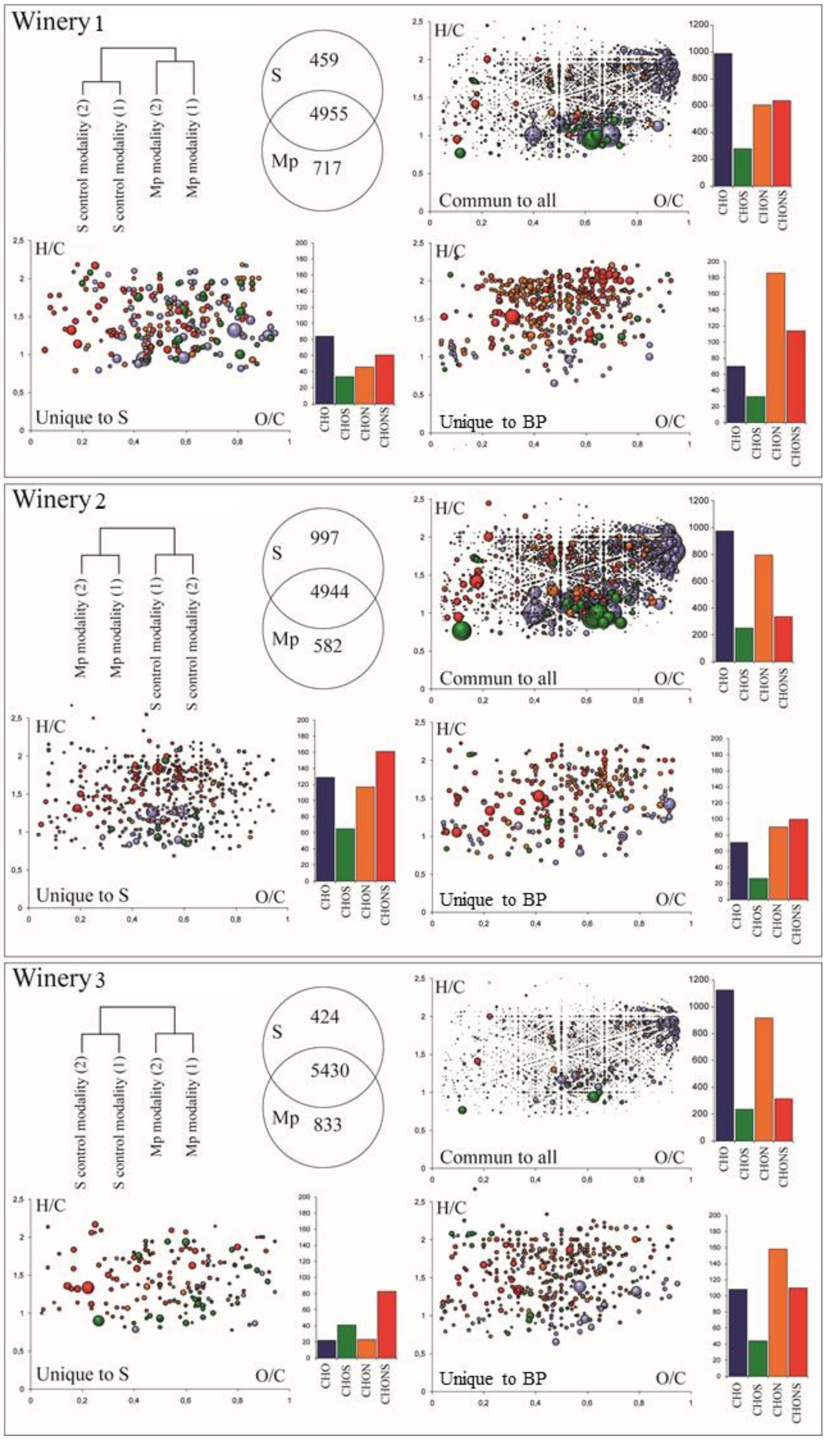

Supplementary Figure 4. (Fig S4) HCA and Venn diagrams representing annotated compounds between BP and S modalities in each winery (1.2 and 3). Van Krevelen diagram (H/C vs. O/C atomic ratio) and histogram (CHO, CHOS, CHON and CHONS) representing all common compounds, unique to S and Unique to BP in each winery (1, 2 and 3).

1.2 Supplementary Tables

Modalities	*L*	*a*	*b*	*Color*	*Overall colourimetric difference: ΔE*
Winery 1 – BP modality	74.30	34.17	-3.48		Yes
Winery 1 – S modality	73.14	35.28	-3.37		
Winery 2 – BP modality	76.44	22.68	1.82		Yes
Winery 2 – S modality	83.03	18.72	0.07		
Winery 3 – BP modality	83.99	18.58	-0.47		Yes
Winery 3 – S modality	86.01	15.22	-0.69		

Supplementary Table 1. (Tab S1) Tristimulus coordinates (L*a*b*) at the end of MLF for BP and S modalities of each winery. L corresponds to lightness/darkness, a to red/green chromaticity, b to yellow/blue chromaticity. Overall colorimetric difference: $\Delta E=((\Delta L)^2+(\Delta a)^2+(\Delta b)^2)^{1/2}$. $\Delta E>2$: Significant color differences (reference: OIV-MA-AS2-11 method).

Compounds (µg/L)	*Winery 1 – BP modality*	*Winery 1 – S modality*	*Winery 2 – BP modality*	*Winery 2 – S modality*	*Winery 3 – BP modality*	*Winery 3 – S modality*
ETHYL ACETATE	**74.15 b**	**107.46 a**	**89.13 ab**	**78.49 b**	**87.18 b**	**78.94 b**
ETHYL ISOBUTYRATE	**0.82 a**	**0.72 a**	**0.46 a**	**0.69 a**	**0.73 a**	**0.62 a**
2.3-BUTANEDIONE	**807.07 a**	**300.89 b**	**466.54 b**	**326.16 b**	**500.15 b**	**309.39 b**
ETHYL BUTYRATE	**4.8 a**	**5.62 a**	**4.1 a**	**4.07 a**	**4.65 a**	**5.57 a**
1-PROPANOL	**1428.37 c**	**1162.43 c**	**2176.43 b**	**1398.58 c**	**2782.3 a**	**976.87 c**
ETHYL VALERATE	**0.46 a**	**0.54 a**	**0.40 a**	**0.38 a**	**0.57 a**	**0.47 a**
ISOBUTYL ALCOHOL	**17652.48 bc**	**21643.66 b**	**34132.06 a**	**33574.18 a**	**15858.57 c**	**15927.16 c**
ISOAMYL ACETATE	**18.33 b**	**36.04 a**	**15.12 b**	**19.54 b**	**7.55 c**	**16.65 b**
1-BUTANOL	**1154.57 a**	**1587.12 a**	**197.62 b**	**178.21 b**	**167.37 b**	**191.74 b**
ETHYL 2-BUTENOATE	**0.17 a**	**0.27 a**	**0.20 a**	**0.19 a**	**0.15 a**	**0.28 a**
3-METHYLBUTANOL	**59376.17 ab**	**73634.04 a**	**60378.89 ab**	**60872.75 ab**	**48692.69 c**	**51743.29 c**
2-METHYLBUTANOL	**381384.14 a**	**423140.02 a**	**239344.84 b**	**265568.20 b**	**231058.81 b**	**236318.31 b**
ETHYL HEXANOATE	**1842.48 ab**	**2221.82 a**	**1450.03 b**	**1454 b**	**1792.43 ab**	**1586.91 b**
HEXYL ACETATE	**1.93 c**	**4.98 b**	**8.35 a**	**4.23 b**	**1.53 c**	**1.40 c**
ETHYL LACTATE	**80.69 cd**	**70.78 d**	**112.94 ab**	**100.03 abc**	**122.15 a**	**86.96 bcd**
1-HEXANOL	**6193.94 a**	**7489.11 a**	**8276.62 a**	**8212.93 a**	**7550.38 a**	**6687.44 a**
ETHYL OCTANOATE	**283.18 b**	**624.08 a**	**250.87 b**	**271.41 b**	**234.7 b**	**232.21 b**
1-OCTEN-3-OL	**59.36 ab**	**57.98 ab**	**59.32 ab**	**45.42 b**	**71.98 a**	**42.11 b**
1-HEPTANOL	**392.06 b**	**410.5 ab**	**394.74 b**	**337.71 b**	**518.89 a**	**380.68 b**
BENZALDEHYDE	**3495.72 c**	**3781.11 c**	**7126.29 b**	**7016.86 b**	**10141.54 a**	**6409.11 a**
ETHYL NONANOATE	**2.43 c**	**4.37 a**	**2.81 c**	**2.96 bc**	**3.12 bc**	**4.01 ab**
1-OCTANOL	**1138.96 a**	**1019.87 a**	**1292.04 a**	**1045.83 a**	**1154.46 a**	**1021.04 a**
TERPINENE-4-OL	**69.74 a**	**90.64 a**	**68.87 a**	**129.03 a**	**170.55 a**	**77.62 a**
ETHYL DECANOATE	**5.37 b**	**17.73 a**	**12.76 a**	**15.32 a**	**6.2 b**	**4.8 b**
GAMMA-BUTYROLACTONE	**0.46 ab**	**0.55 a**	**0.22 c**	**0.25 bc**	**0.41 abc**	**0.33 bc**
NONANOL	**658.51 b**	**775.21 b**	**1774.83 a**	**1872.91 a**	**1016.7 b**	**1108.62 b**
ISOAMYL OCTANOATE	**0.26 b**	**0.63 ab**	**0.90 a**	**0.99 a**	**0.69 ab**	**0.31 b**
ACETOPHENONE	**69.82 a**	**68.71 a**	**29.01 b**	**28.49 b**	**28.05 b**	**22.63 b**
DIETHYL SUCCINATE	**3.5 c**	**4.57 c**	**3.63 c**	**7.53 ab**	**8.34 a**	**6.61 b**
PHENYL ETHYL ACETATE	**0.80 b**	**1.19 a**	**0.7 bc**	**0.65 bc**	**0.47 bc**	**0.43 c**
TRANS-BETA-DAMASCENONE	**0.32 a**	**0.4 a**	**0.42 a**	**0.44 a**	**0.42 a**	**0.3 a**
ETHYL LAURATE	**1.52 b**	**1.94 b**	**4.19 a**	**4.02 a**	**1.39 b**	**2.26 b**
BENZYL ALCOHOL	**732.92 a**	**876.07 a**	**554.28 a**	**670.95 a**	**635.16 a**	**718.17 a**
PHENYLETHYL ALCOHOL	**118789.2 a**	**102020.51 b**	**70391.13 cd**	**78044.84 c**	**68850.13 cd**	**62302.64 d**
ETHYL MYRISTATE	**4.69 ab**	**5.44 a**	**3.37 b**	**3.43 b**	**3.69 ab**	**5.37 a**
ETHYL PALMITATE	**2.05 ab**	**2.71 a**	**0.74 c**	**0.77 bc**	**1.31 bc**	**1.54 abc**
HEXANOIC ACID	**4016.62 b**	**4600.97 b**	**10894.75 a**	**12064.12 a**	**8522.91 ab**	**11468.32 a**
OCTANOIC ACID	**5344.44 ab**	**7037.92 a**	**4203.29 b**	**4851.06 b**	**4830.04 b**	**4815.67 b**
DECANOIC ACID	**484.05 a**	**675.35 a**	**610.82 a**	**538.89 a**	**471.49 a**	**450.27 a**

Supplementary Table 2. (Tab S2) Concentrations and univariate statistical analysis of the quantified volatile compounds (µg/L) for Mp and S control modalities in Winery 1, 2 and 3.
